# Attitude towards dengue control efforts with the potential of digital technology during COVID-19: partial least squares-structural equation modeling

**DOI:** 10.12688/f1000research.125318.1

**Published:** 2022-11-10

**Authors:** Sang Gede Purnama, Dewi Susanna, Umar Fahmi Achmadi, Tris Eryando, Luh Putu Lila Wulandari

**Affiliations:** 1 Doctoral Study Program, Faculty of Public Health, Universitas Indonesia, Depok, Jawa Barat, 16424, Indonesia; 2 Department of Public Health and Preventive Medicine, Medicine Faculty, Udayana University, Denpasar, Bali, Indonesia; 3 Department of Environmetal Health, Faculty of Public Health, Universitas Indonesia, Depok, Jawa Barat, 16424, Indonesia; 4 Department of Biostatistics and Population Studies, Faculty of Public Health, Universitas Indonesia, Depok, Jawa Barat, 16424, Indonesia; 5 The Kirby Institute, University of New South Wales, Sydney, New South Wales Email, Australia

**Keywords:** attitude, dengue, potential, technology, modelling dengue transmission

## Abstract

**Background: **Dengue fever is still a public health issue in Indonesia, and during the coronavirus disease 2019 (COVID-19) pandemic, integrated digital technology will be required for its control. This study aimed to identify the factors that influence attitudes toward dengue control concerning the potential application of digital technology.

**Methods: **This was a cross-sectional survey, with 515 people willing to fill out an online questionnaire. The analysis was conducted using Partial Least Square-Structural Equation Modelling (PLS-SEM). There were 46 indicators used to assess attitudes toward dengue control, which are organized into six variables: the need for digital information systems, perceptions of being threatened with dengue, the benefits of dengue control programs, program constraints, environmental factors and attitudes in dengue control.

**Results: ** The source of information needed for dengue control was mainly through social media. There was a positive relationship between perception of environmental factors to perception of dengue threat, perception of program constraints, perception of program benefits, and perception of digital technology needs. Perception of program benefits and threatened perception of dengue have a positive relationship with perception of digital technology needs.

**Conclusions: **This model showed the variables perception of digital technology and perception of benefits had a positive association with attitude towards dengue control.

## Introduction

Dengue hemorrhagic fever (DHF) is still a global public health problem in tropical and subtropical climates. This mosquito-borne disease has spread rapidly in the last 50 years, and WHO estimates that the annual cases reach 50–100 million DHF infections. ^1^ Furthermore, the cases have tripled to 390 million, with more than 70% of the world’s population at risk. ^2^

The global spread of dengue fever is influenced by urbanization, globalization, and less effective vector control. The level of dense human population in an area is also followed by the density level of the Aedes aegypti mosquito. ^3^^–^^5^ The hemorrhagic fever can be transmitted through mosquito bites from one human to another. In addition, the development of the aviation industry in various countries increases the mobility of humans and vectors from one country to another. The lack of practical control efforts has led to dengue disease outbreaks in various regions. ^6^

More than 70% of the population at risk of DHF live in the Southeast Asia and West Pacific region, with a global disease burden of 75%. Therefore, WHO promotes making strategic plans to quickly detect and control disease outbreaks and stop their spread to new areas. ^7^ Sustainable vector control methods, public health policymakers, and vaccine development should receive serious attention in controlling the current and future global distribution of DHF. ^8^

Indonesia is one of the countries endemic to dengue fever. The first DHF case was reported in 1968 in Surabaya, and since then, the incidence rate has increased from 0.05 to 35-40 per 100,000 population and peaked in 2010 (IR 85). ^9^ Based on the Ministry of Health report, until July 2020, there were 73,329 cases and 467 deaths. The regencies with the highest incidence rates in 2020 are Buleleng, Bali (2677 cases), Badung, Bali (2,138 cases), Bandung City (1,748 cases), East Jakarta (1,765 cases), and Sikka (1,715). ^10^

During the current coronavirus disease 2019 (COVID-19) pandemic, efforts to control DHF cannot be carried out optimally because of health protocols. These include social distancing, wearing masks, and being careful about receiving foreign guests. This makes it challenging to collect data door to door, and the condition requires a digital technology approach to conduct surveillance and health education in the community. An integrated dengue surveillance and control system is needed in the endemic areas. Data collection should be quick and easy, as well as educate the public on vector control. Therefore, it is necessary to study the potential development of digital technology in dengue control during the COVID-19 pandemic.

## Methods

### Conceptual model

**Figure 1.**
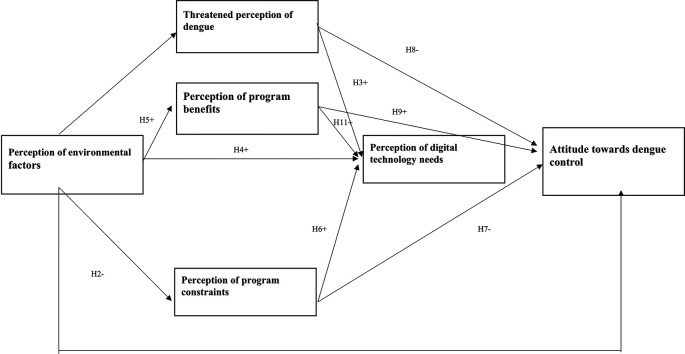
The structural hypothesis of the relationship between perception and attitude in dengue. ^14^

The theoretical model adopts a health belief model between perceptions and dengue control behaviour. ^11^^–^^13^ The health belief theory is then modified by adding environmental variables and the need for digital technology. The hypotheses were compared with six latent constructs related to dengue control attitudes, influenced by perceptions of the threat of dengue, program benefits, environmental factors, program constraints, and technology needs ( Figure 1). The direction of the path shows the (+) and (-) effects of the relationship, and this study assessed the accuracy of the model and hypothesis with PLS-SEM.

Regarding potential bias in this study, online data collection means that respondents can answer questions repeatedly. Thus, to reduce bias, data validation was carried out based on names and addresses. Incomplete answers, this is done with a re-checking system and requires answering. Respondents also only represented the Denpasar City area, not representing Indonesia.

### Study design and data collection

This cross-sectional study is conducted using an online survey with 6 variables. ^15^ These include perceptions of the need for digital information systems, dangers of DHF, benefits of DHF control programs, program constraints, and environmental factors related to attitudes toward controlling DHF. Respondents answered with a Likert scale of 1-5, where 1, 2 3, 4, and 5 represent strongly disagree, disagree, neutral, agree, and strongly agree. The questionnaire was made by discussing with experts and testing about 30 respondents to measure the validity and reliability. Respondents were selected based on inclusion criteria, aged more than 17 years, having an address in Denpasar City for more than one year, and willing to answer questions. The results of the validity and reliability tests found that 46 of the indicators were declared valid. Invalid indicators are excluded and not used. The final questionnaire can be found as *Extended data*. ^15^

It was then distributed online using a google form, and data collection was carried out in the Denpasar City area, which is endemic to DHF. Table 1 shows a description of the data from the composites and indicators, as well as the definitions of attitudes towards dengue control efforts with the other five composites.

**Table 1.** Descriptive data.

Composite	Indicator	Definition	
Perception of program benefits	Var1a	*Jumantik* volunteers always visit my house every month	
	Var1b	*Jumantik* volunteers always provide information	
	Var1c	J *umantik* volunteers give larvicide	
	Var1d	*Jumantik* program is useful for preventing dengue	
	Var1e	Students can play the role of being a larva care student	
	Var1f	I support the dengue control program	
	Var1g	I am willing to follow *Jumantik*’s advice	
Perception of being threatened with dengue	Var2a [Table-fn tfn1]	I am at risk of being infected with dengue	
	Var2b *	My family is at risk for dengue infection	
	Var2c	Dengue Haemorrhagic Fever (DHF) is a deadly disease	
	Var2d *	Dengue is a highly contagious disease	
	Var2e	Dengue Haemorrhagic Fever (DHF) is a dangerous disease	
	Var2f	We are afraid of being infected with dengue	
Perception of program constraints	Var3a	Program funding is still lacking
	Var3b	A limited number of health workers	
	Var3c	Home visits are limited due to social distancing and COVID-19	
	Var3d	Visiting hours during business hours from 8 to 10 AM	
	Var3e *	Limited information	
	Var3f *	Limited larvicides	
	Var3g *	Brochure distribution is rarely done	
	Var3h *	Limited smartphone facilities	
Perception of digital technology needs	Var4a	I am willing to use my cell phone for the dengue control program	
	Var4b	I am willing to fill in the data on the website	
	Var4c	I am willing to watch digital educational videos	
	Var4d	I am willing to share information with my family	
	Var4e	Support dengue control digital information system	
	Var4f	I have an android phone that supports the program	
	Var4g	I have social media applications such as WhatsApp, Facebook, Instagram, and others	
	Var4h *	Usually, use WhatsApp to communicate	
Perception of environmental factors	Var5a	The rainy season affects the incidence of dengue	
	Var5b	The number of water containers affects mosquito density	
	Var5c *	Aedes mosquitoes like to lay their eggs in clean water	
	Var5d	A bucket filled with water has the potential to become mosquito breeding place	
	Var5e	Bath containers have the potential to become mosquito breeding places	
	Var5f	Used bottles, used tires can become mosquito breeding places	
	Var5g	Empty land has the potential to become mosquito breeding places	
	Var5h	Environmental conditions affect dengue cases	
Attitude towards dengue control	Var6a	I am willing to eradicate mosquito breeding places once a week	
	Var6b	I am willing to close the water container	
	Var6c	Carry out environmental cleaning activities once a week	
	Var6d	Fill in larva density data every week	
	Var6e	Support the program to eradicate mosquito breeding places	
	Var6f	Support the activities of students caring for dengue every week	
	Var6g *	I am willing to be penalized if larvae are found	
	Var6h *	I am willing to pay a fine if a larva is found	
	Var6i	Willing to make efforts to eradicate mosquito breeding places following the advice of the officer	

* These indicators were not included in latent variables due to the multicollinearity criteria of PLS-SEM.

*Jumantik* is a volunteer recruited from each village area to inspect, monitor, and control dengue vectors. They were given the task of conducting daily inspections to visit homes. The results of their activities are reported as vector entomological surveillance. This is part of community empowerment to carry out dengue control in their area actively.

### Sample

The inclusion criteria were respondents who were over 17 years old and had resided in Denpasar City for more than six months. They are willing to fill out a research approval form and receive mobile phone credit from the internet provider for two. Even though 596 respondents filled in the data, only 515 fulfilled the requirements and were complete. Sampling was carried out with non-random sampling conducted online in a limited population with the consideration that respondents could not be visited directly due to the COVID-19 pandemic in the Denpasar City area, which had previously been permitted by the Licensing Service, Health Service, Head of Public Health Center, and Village Head.

### Variables

This study consists of six variables with 46 indicators using a Likert scale of 1-5, where 1, 2, 3, 4, and 5 representing strongly disagree, disagree, neutral, agree, and strongly agree. Attitudes toward prevention strategies are a dependent variable that tends to act to regulate dengue in the surrounding environment through the use of vector control activities at breeding sites for mosquitoes. Therefore, nine indicators are measured, namely willingness to carry out a weekly movement to eradicate mosquito breeding areas, close water reservoirs, clean the environment regularly, filling in data on larval density weekly independently, providing assistance to dengue control programs, supporting students’ weekly larvae care activities, willing to be sanctioned when larvae are discovered, willing to pay a fine, and making efforts to eradicate mosquito breeding areas following the officer’s advice.

Perceptions of the benefits are related to the assessment of dengue control programs beneficial to the community. These consist of 7 indicators: *jumantik* volunteers always visit every month, the officers always provide information, the volunteers provide larvacide, the program is useful for preventing dengue cases, and students play a role for larvae, the dengue control program was supported, and the officer’s advice was followed.

The perception of being threatened with dengue is a condition that causes feelings of fear and vulnerability to outbreaks which consist of 6 variables. These include the risk of being infected with dengue fever and several families at risk of being infected. Dengue fever is a deadly, easily contagious, and dangerous disease that people are afraid of being infected.

Perceptions of program constraints are obstacles in carrying out activities related to facilities and pandemic conditions in dengue control. These consist of 8 variables of limited program funding, *jumantik* personnel, home visit activities due to social distancing and COVID-19, visiting time, which is during working hours from 8 to 10, the information provided, the larvicides, and smartphone facilities.

The need for digital technology is a public perception of the support for implementing the systems in dengue control. These consist of 8 variables, namely being willing to use mobile phones for dengue control programs, filling in data on websites, watching digital educational videos, sharing information with family, supporting digital information system programs, having Android phones that support the program, having social media applications such as WhatsApp, Facebook, Instagram, and others, but accustomed to using WhatsApp to communicate.

Perception of environmental factors is the surrounding conditions that affect the density of larvae and dengue cases, both natural and artificial. These consist of 8 variables, namely the rainy season affects the incidence of dengue, the number of water reservoirs affects the density of mosquitoes, the Aedes mosquitoes lay eggs in clean water, the bucket filled with water in bathroom containers, used bottles, tires, and vacant places have the potential to become a breeding place.

### Data analysis

This study was analyzed using PLS-SEM with SmartPLS 3.0 software. It analyzed five variables related to attitudes towards dengue control. The PLS-SEM analysis uses two stages, and the first describes the measurement model connecting the constructs and indicators to the theory. In the second stage, the structural model determines the determinants of the relationship between the construction and the hypothetical model.

### Ethical approval

This study is part of a research carried out for the development of an integrated dengue control system. This study has been approved by the ethics committee of the Faculty of Public Health, University of Indonesia (Ket-416/UN2.F10. D11/PPM.00.02/2021). Before data collection, informants had received information about their goals, risks, and rights. In addition, a written consent form was given before the interview, and all information from participants is confidential and for this study only.

## Results

Table 2 shows the socio-demographics of respondents who filled in the data for this study. There were 515 respondents, with 41.4% and 58.6% being men and women, respectively, with the highest education level being high school level with 62.3%. The respondents’ age distribution was mainly 40-44 years old (19.4%) and 17-24 years (18.6%). The type of occupation was primarily private workers (29.3%) and housewives (17.9%).

**Table 2.** Socio-demographic of respondents.

Respondent characteristics	Frequency	Percent
Gender		
Male	213	41.4
Female	302	58.6
Education		
Primary School	2	0.4
Junior High School	27	5.2
Senior High School	321	62.3
Diploma	59	11.5
Bachelor	106	20.6
Age (years)		
17-24	96	18.6
25-29	28	5.4
30-34	56	10.9
35-39	64	12.4
40-44	100	19.4
45-49	79	15.3
50-54	44	8.5
55-59	28	5.4
60	20	3.9
Occupation		
Unemployment	31	6
Civil servant	27	5.2
Health worker	58	11.3
Housewife	92	17.9
College student	86	16.7
Village head	15	2.9
Entrepreneur	48	9.3
Private sector employee	151	29.3
Teacher	7	1.4

**Figure 2.**
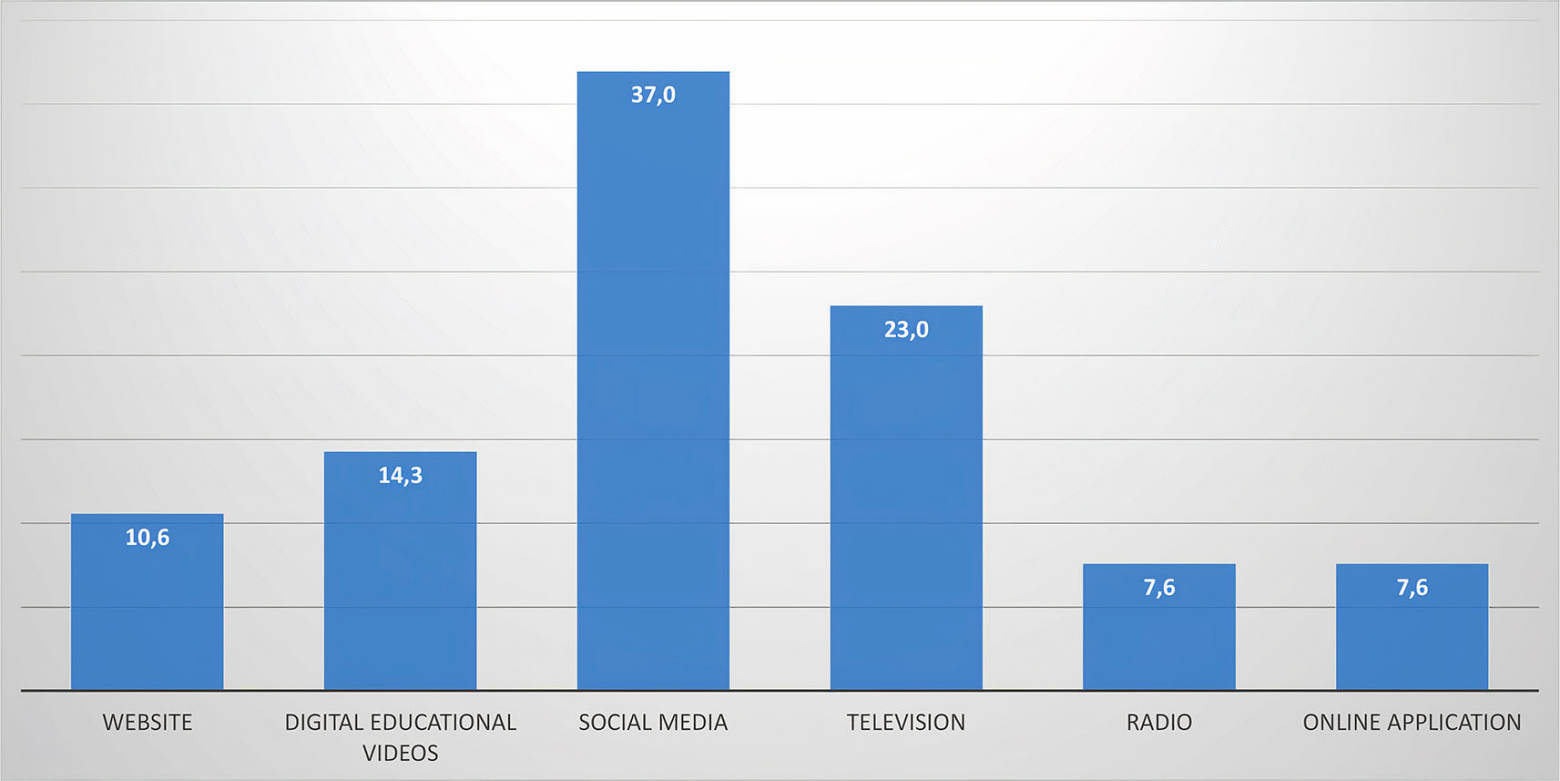
Percentage of sources of information technology needed regarding dengue control. ^14^

Most sources of information used for dengue control are through social media such as WhatsApp, Facebook, Instagram, Tiktok, and others (37%). Most respondents find it easier to get information through social media. However, there has been a change in the sources due to the development of information technology. Another highest source of knowledge is television (23%), followed by digital educational videos (14.3%) and websites (10.6%) ( Figure 2).

**Figure 3.**
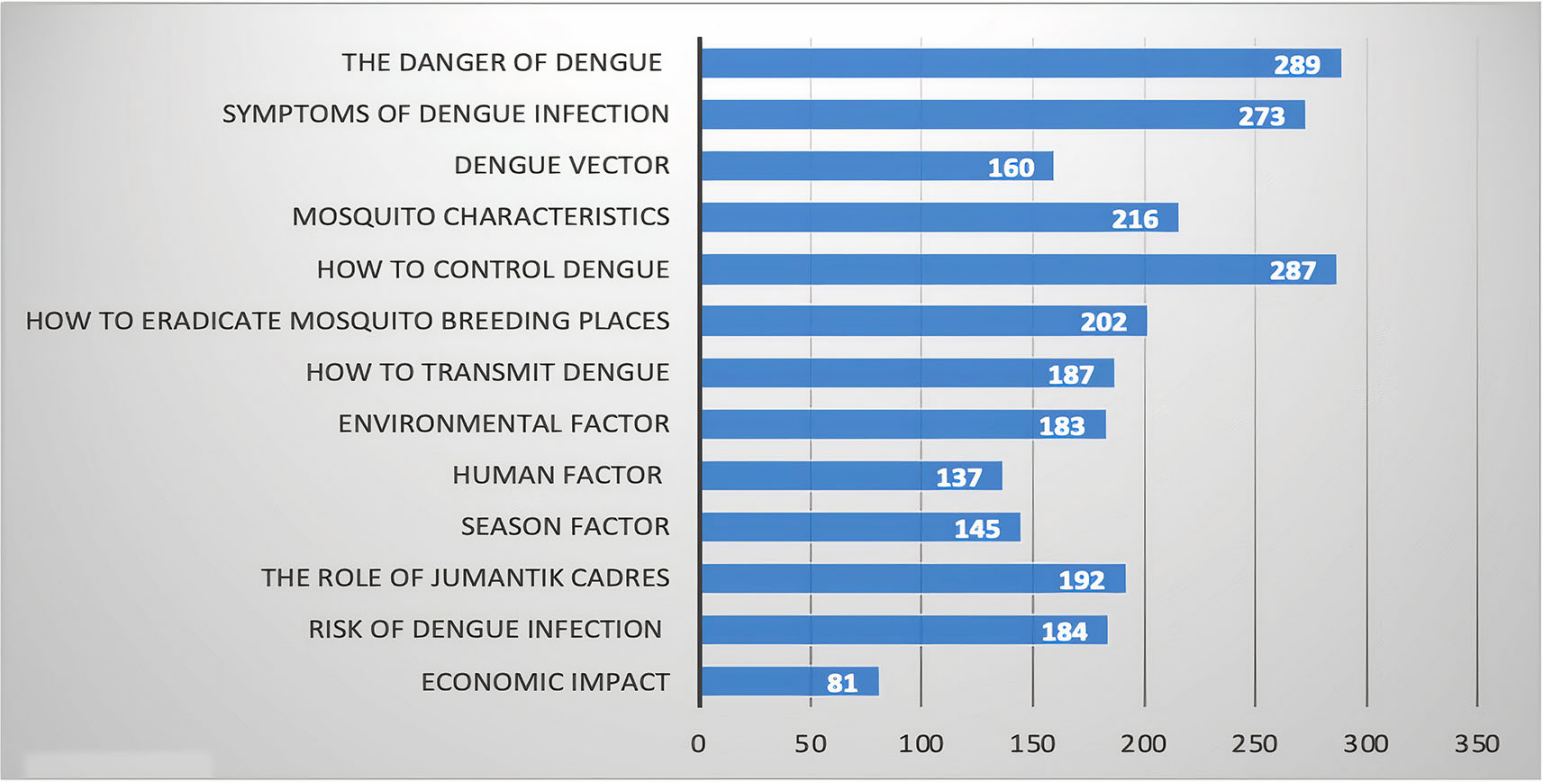
Types of information needed in dengue control. ^14^

The types of information needed are the methods of controlling dengue, the dangers, symptoms of infection, characteristics of dengue-transmitting mosquitoes, the risk, the role of *jumantik* cadres, methods of eradicating mosquito breeding sites, and environmental factors. This information is needed to develop digital educational media for dengue control ( Figure 3).

**Figure 4.**
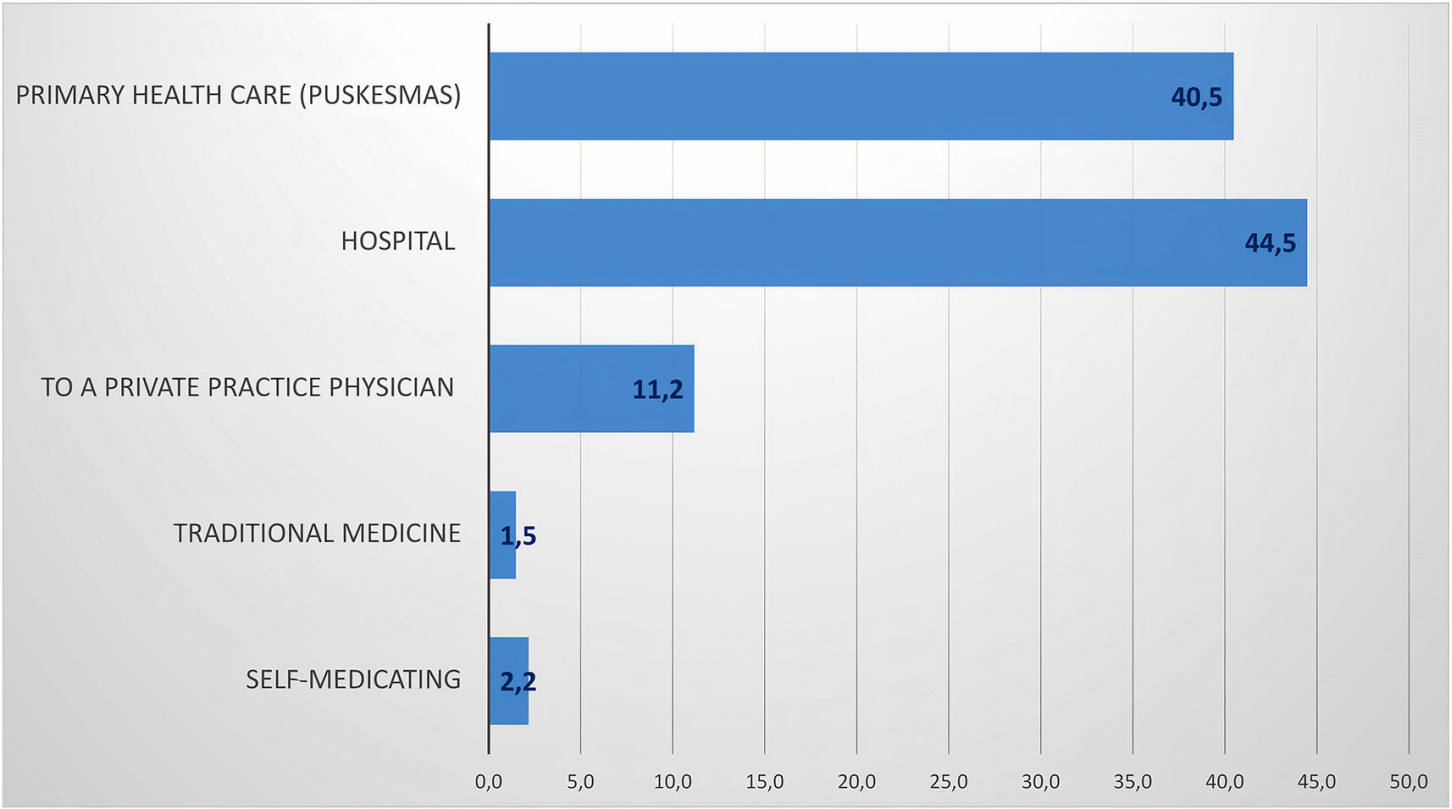
Percentage of seeking health services if infected with dengue. ^14^

The percentage of the community’s efforts to seek health services in handling dengue symptoms is through hospitals (44.5%) and primary health centers (40.5%). Public awareness to conduct health checks while experiencing symptoms of DHF is high in the urban setting in which the number and proximity of health-care services are relatively close and easily accessible ( Figure 4).

This section details the results obtained for the proposed study model.

### Measurement model

*Composite mode A*

**Figure 5.**
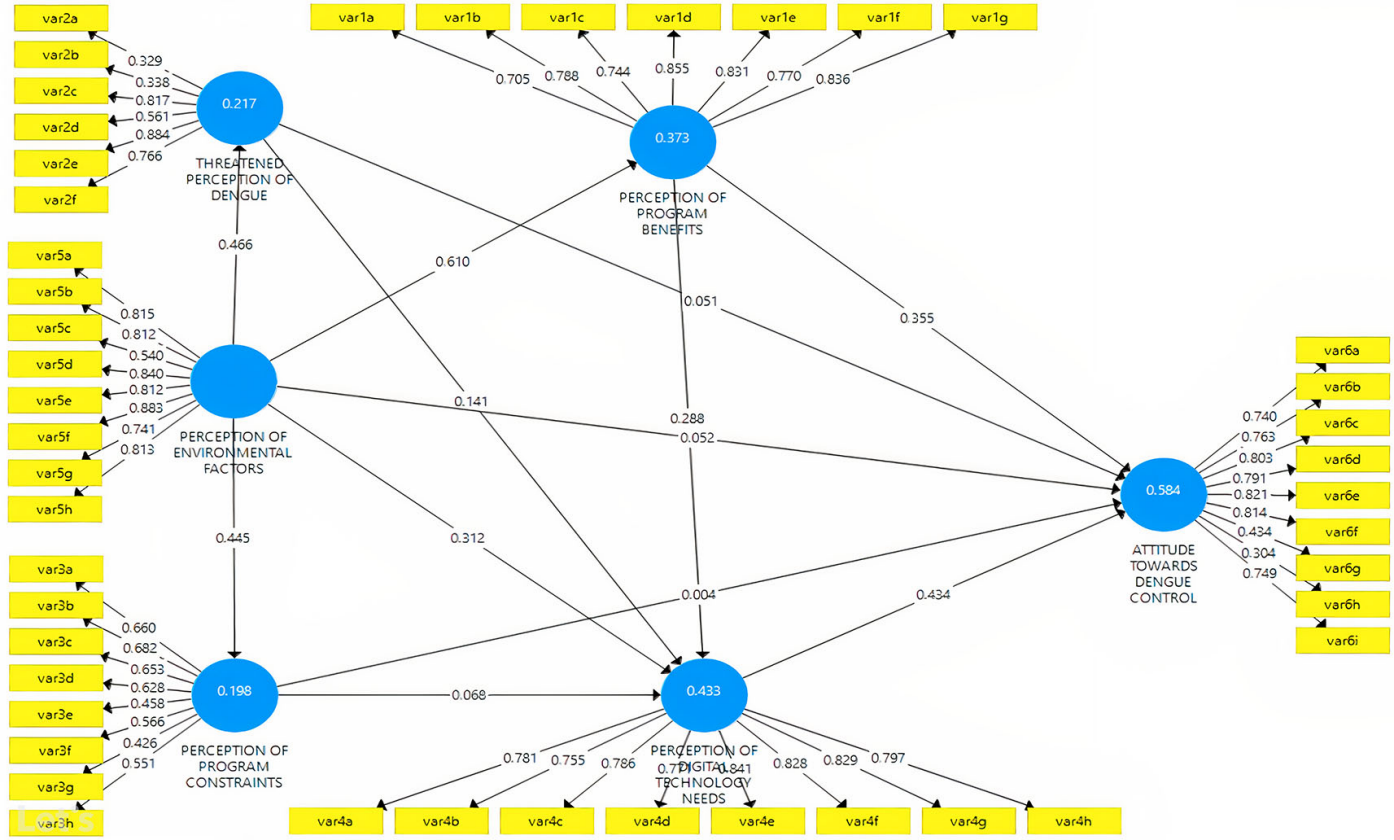
Study model. ^14^

The composite measurement model in mode A (attitude) was assessed in individual item reliability, construct reliability, convergent validity, and discriminant validity. First, the reliability of each item is analyzed through a loading factor, as seen in Figure 5.

Table 3 shows the value of the measurement of validity and reliability. Cronbach’s Alpha value and composite were used to evaluate construct reliability. The values show that the construct exceeds the recommended cut-off of 0.7. Convergent validity was also proved because the construct’s extracted mean-variance (AVE) was higher than 0.500. Table 3 shows that the measurement model meets the criteria.

**Table 3.** Validity and reliability measurement.

Composite	Cronbach’s Alpha	Composite Reliability (CR)	Average Variance Extracted (AVE)
Attitude Toward Dengue Control	0.901	0.921	0.627

Table 4 presents discriminant validity results through the heterotrait-monotrait (HTMT) correlation ratio. All constructs reach discriminant validity because the confidence interval does not contain a zero value. This situation means that each variable is different from one another. The data examined in the measurement model show that the attitude construct measure is reliable and valid.

**Table 4.** Heterotrait-monotrait (HTMT) inference.

HTMT inference *	Original sample	Sample mean	5%	95%
Perception Of Digital Technology Needs -> Attitude Towards Dengue Control	0.733	0.735	0.657	0.804
Perception Of Environmental Factors -> Attitude Towards Dengue Control	0.596	0.595	0.476	0.708
Perception Of Program Benefits -> Attitude Towards Dengue Control	0.714	0.717	0.637	0.793
Perception Of Program Constraints -> Attitude Towards Dengue Control	0.456	0.450	0.320	0.564
Threatened Perception Of Dengue -> Attitude Towards Dengue Control	0.486	0.484	0.358	0.611

* Significance, the confidence interval 95% bias was corrected and performed using bootstrap procedure with 10,000 replications.

*Composite mode B*

**Figure 6.**
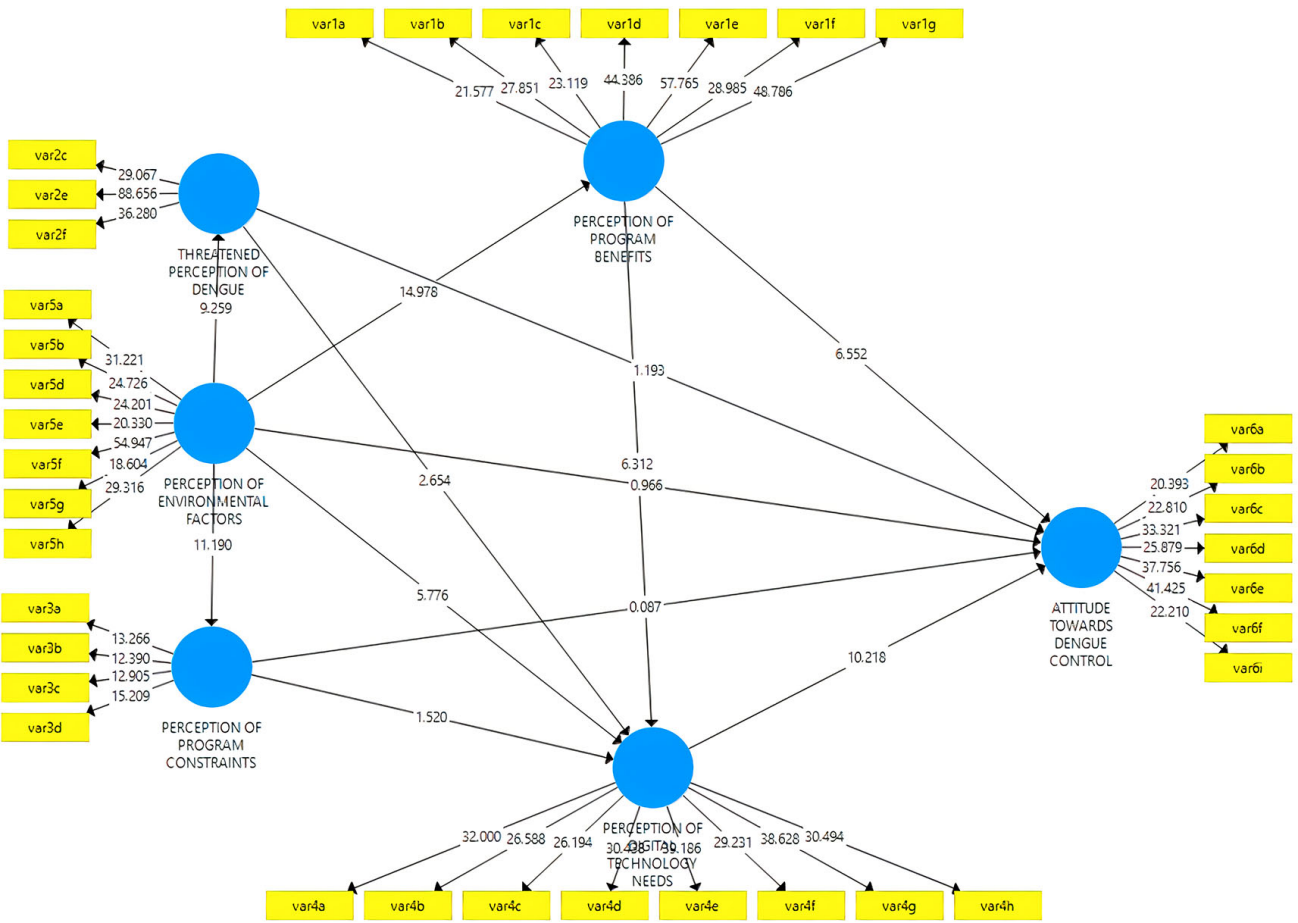
Model results SEM-PLS. ^14^

The composite measurement model in mode B was assessed in collinearity between the outer weights’ indicators, significance, and relevance. First, removing the indicator is carried out when the value exceeds the variance impact factor (VIF = 3). As a result of this process, only the indicators shown in Table 1 are without collinearity. Second, the relevance of the weights is analyzed, and Figure 6 shows the indicators in construction for latent variables. Finally, it is possible to start a bootstrap with 10,000 sub-samples to assess significance. Indicators with insignificant weights but significant loadings of 0.50 or higher were considered relevant ( Table 5).

**Table 5.** Significance of weights.

	Original sample (O) *	t	Loading	Lo95	Hi95
**Perception of program benefits**					
Var1a	0.124	14.562	0.702	0.106	0.141
Var1b	0.142	17.507	0.786	0.125	0.156
Var1c	0.139	12.786	0.742	0.117	0.159
Var1d	0.181	22.076	0.855	0.166	0.199
Var1e	0.218	16.247	0.832	0.193	0.247
Var1f	0.217	16.050	0.772	0.193	0.244
Var1g	0.232	20.284	0.837	0.211	0.255
**Threatened perception of dengue**					
Var2c	0.364	16.095	0.831	0.319	0.407
Var2e	0.438	19.056	0.910	0.400	0.490
Var2f	0.364	13.604	0.820	0.306	0.412
**Perception of program constraints**					
Var3a	0.326	6.585	0.670	0.239	0.429
Var3b	0.265	6.616	0.666	0.188	0.339
Var3c	0.391	8.735	0.682	0.297	0.472
Var3d	0.479	8.557	0.706	0.382	0.600
**Perception of digital technology needs**					
Var4a	0.154	18.282	0.780	0.138	0.171
Var4b	0.132	17.528	0.752	0.118	0.146
Var4c	0.142	16.837	0.784	0.125	0.158
Var4d	0.182	20.493	0.774	0.165	0.201
Var4e	0.164	23.472	0.841	0.150	0.177
Var4f	0.158	17.940	0.828	0.141	0.175
Var4g	0.163	19.850	0.830	0.149	0.181
**Perception of environmental factors**					
Var5a	0.201	19.233	0.827	0.183	0.222
Var5b	0.167	17.922	0.820	0.150	0.187
Var5d	0.163	17.488	0.831	0.146	0.181
Var5e	0.158	17.032	0.804	0.139	0.176
Var5f	0.188	20.754	0.887	0.172	0.207
Var5g	0.160	17.451	0.745	0.145	0.181
Var5h	0.181	20.064	0.823	0.165	0.201
**Attitude towards dengue control**					
Var6a	0.163	21.560	0.753	0.149	0.179
Var6b	0.169	18.937	0.779	0.151	0.186
Var6c	0.171	19.730	0.815	0.157	0.189
Var6d	0.166	20.917	0.778	0.151	0.182
Var6e	0.202	23.747	0.835	0.188	0.220
Var6f	0.197	21.106	0.823	0.179	0.216
Var6i	0.194	18.306	0.754	0.175	0.215

* t statistic, and 95% bias-corrected confidence interval performed by a bootstrapping procedure with 10,000 replications.

*Structural model*

The structural model is evaluated after verifying the correctness of the construction measurements. The path coefficients and their 10,000 resampling bootstrap significance levels are reported in Table 6 and Figure 6. Additionally, Table 6 shows that the VIF constructs range from 1,000 to 1,700, indicating no collinearity between variables. This study also assesses the quality by examining the overall predictive relevance of the model with a Q2 value above zero which indicates a fit in the prediction model. The magnitude of Q2 has a value of 0 < Q2 < 1, where the closer to 1, the better the model. The coefficient of determination (R2) also exceeds 0.1 for endogenous latent variables since the construct has an acceptable predictive power quality.

**Table 6.** Whole sample results.

Direct effect	Path	t	p	Lo95	Hi95	f ^2^	VIF
Perception of Environmental Factors -> Threatened Perception of Dengue	0.478	9.259	0.000	0.378	0.583	0.296	1,000
**R ^2^=0.227**							
Perception of Environmental Factors -> Perception of Program Constraints	0.471	11.190	0.000	0.392	0.553	0.285	1,000
**R ^2^=0.220**							
Perception of Environmental Factors -> Perception of Program Benefit	0.606	14.978	0.000	0.532	0.689	0.580	1,000
**R ^2^=0.366**							
Perception of Environmental Factors -> Perception of Digital Technology Needs	0.322	5.776	0.000	0.206	0.422	0.100	1,993
Perception of Program Benefits -> Perception of Digital Technology Needs	0.293	6.312	0.000	0.198	0.380	0.087	1,862
Perception of Program Constraints -> Perception of Digital Technology Needs	0.062	1.520	0.129 ^ns^	-0.020	0.138	0.005	1,328
Threatened Perception of Dengue -> Perception of Digital Technology Needs	0.120	2.654	0.008	0.035	0.214	0.018	1,395
**R ^2^=0.427**							
Perception of Digital Technology Needs -> Attitude Towards Dengue Control	0.405	10.218	0.000	0.333	0.479	0.219	1,765
Perception of Environmental Factors -> Attitude Towards Dengue Control	0.062	0.966	0.335 ^ns^	-0.058	0.187	0.005	1,993
Perception of Program Benefits -> Attitude Towards Dengue Control	0.371	6.552	0.000	0.271	0.488	0.172	1,862
Perception of Program Constraints -> Attitude Towards Dengue Control	0.003	0.087	0.930 ^ns^	-0.057	0.064	0.000	1,328
Threatened Perception of Dengue -> Attitude Towards Dengue Control	0.050	1.193	0.234 ^ns^	-0.042	0.127	0.004	1,430
**R ^2^=0.571, Q ^2^=0.569**							

Indirect Effect	Path	t	p	Lo95	Hi95	VAF	VIF
Perception of Environmental Factors -> Perception of Digital Technology Needs-->Attitude Towards Dengue Control	0.487	10.269	0.000	0.398	0.587	0.511	na
Perception of Environmental Factors -> Threatened Perception of Dengue-->Attitude Towards Dengue Control	0.487	10.269	0.000	0.398	0.587	0.752	na
Perception of Environmental Factors -> Perception of Program Benefits-->Attitude Towards Dengue Control	0.487	10.269	0.000	0.398	0.587	0.469	na
Perception of Environmental Factors ->Perception of Program Benefits--> Perception of Digital Technology Needs	0.264	6.938	0.000	0.198	0.350	0.300	na
Perception of Program Benefits -> Perception of Digital Technology Needs-->Attitude Towards Dengue Control	0.119	5.267	0.000	0.074	0.165	0.152	na
Perception of Program Constraints -> Perception of Digital Technology Needs-->Attitude Towards Dengue Control	0.025	1.455	0.146 ^ns^	-0.008	0.060	0.058	na
Threatened Perception of Dengue ->Perception of Digital Technology Needs--> Attitude Towards Dengue Control	0.049	2.626	0.009	0.015	0.085	0.097	na

Note: ns= not significant. t statistic, and confidence 95% bias was corrected. The interval was performed using a bootstrap procedure with 10,000 replication. VIF: Inflation of model variance in factors; VAF: variance recorded.

From Table 6, there is a direct influence of Perception of Environmental Factors on the Threatened Perception of Dengue, Program Constraints, Program Benefits, and Digital Technology Needs. Perception of Program Benefits and Threatened Perception of Dengue directly influences Digital Technology Needs. In general, Perception of Digital Technology Needs and Program Benefits directly influence Attitude Towards Dengue Control. Variables Perception of Digital Technology Needs and Perception of Program Benefits positively correlate to Attitude Towards Dengue Control.

VAF values above 80% indicate that the variable serves as a full mediator. The variable can be categorized as a partial mediator when the VAF value ranges from 20% to 80%. However, when the value is less than 20%, it can be concluded that there is almost no mediating effect. The value of VAF indicates that the proportion of Perception Of Digital Technology Needs from the pathway has no mediating effect (VAF<0.2 or 20%). Perception of Digital Technology Needs, Threatened Perception of Dengue, and Program Benefits can be categorized as partial mediators between Environmental Factors and Attitudes Towards Dengue Control (see the indirect effect in Table 6).

## Discussion

The use of digital technology in dengue surveillance is currently needed, specifically during the COVID-19 pandemic. Health protocols such as social and physical distancing make direct door-to-door observation activities difficult. Therefore, there is an increase in smartphones and digital applications in conducting disease surveillance.

This study is a novelty in developing a new model that adopts the health belief model and then collaborates between digital information systems with perceptions of environmental factors, disease threats, and the obstacles related to dengue control attitudes. This study begins with a qualitative study of the potential development of digital surveillance for dengue control, which requires a digitally integrated system for reporting in real-time. ^16^

This study aims to determine the variables that influence attitudes in dengue control related to the potential application of digital technology. It indicates a direct influence of Perception of Environmental Factors on Threatened Perception of Dengue, Program Constraints, Program Benefits, and Digital Technology needs. Perception of Program Benefits and Threatened Perception of Dengue directly Influences Digital Technology Need. Perception of Digital Technology Needs and Program Benefits directly influence Attitude Towards Dengue Control.

Most sources of information needed for dengue control are through social media such as WhatsApp, Facebook, Instagram, Tiktok, and others (37%). Due to the availability of internet access and public WIFI, people have switched their sources of information, which were previously direct from health workers, television, and then using social media. In addition, teenagers use internet media for learning, specifically in urban areas, and access to information is high. ^17^^–^^20^

Attitudes in dengue control are directly influenced by the variable perception of the need for digital technology and program benefits. This is related to the source of information obtained through digital media. Perception of environmental factors is influenced by Threatened Perception of Dengue, Program Constraints, Program benefits, and Digital Technology Need.

Other studies showed an increase in the use of digital technology during the pandemic for monitoring, surveillance, detection, and prevention of COVID-19. ^21^^,^^22^ Studies in Saudi use various digital platforms such as mobile health applications, artificial intelligence, and machine learning in the pandemic surveillance. ^23^ A digital dengue surveillance system has also been developed to predict, detect and control the threat of outbreaks. ^24^^–^^26^ The incidence is often related to climate change, ecological and socio-demographic factors. ^27^^–^^31^ Developing a system based on technology and the environment using spatial mapping makes it possible to predict the potential for outbreaks in an area. ^32^^,^^33^

### Study strengths and limitations

The strength is the development of a model that combines measurement of attitudes towards dengue control with environmental factors on the threatened perception of dengue, program constraints, program benefits, and digital technology needs. The commonly used model is the health belief, but a different approach combines the perceived need for digital technology, environmental factors, and health beliefs.

This study uses PLS-SEM analysis which was selected because it is variance-based and estimates composite components and factors. ^34^ The PLS analysis is a multivariate statistical technique that compares several responses and explanatory variables. ^35^^–^^37^ Through this approach, it is possible to make appropriate structural equations toward dengue control related to the perception of environmental factors on the threatened perception of dengue, program constraints, program benefits, and digital technology needs.

The use of online surveys is limited to certain areas and does not represent the whole of Indonesia, only Denpasar City. Generally, the respondents used were those with mobile phones and internet networks, and they were not randomly assigned.

### Policy implications and future studies

The results are helpful for policymakers to promote the use of digital technology in data collection of disease cases, surveillance, monitoring, and evaluation of health programs supported by socialization through social media that can influence perceptions of the benefits of the program. The community’s attitude toward controlling the disease is also related to the source of information that affects public perception. Policies to support digital facilities such as the availability of internet networks, computer facilities, mobile phones, and data packages affect the disease reporting system and its control. In the future, it is necessary to develop an integrated digital system for reporting disease cases and collecting data on the ecological environment, specifically larval density. This system should perform spatial mapping and predict the potential for a dengue outbreak to occur. Therefore, technology can be helpful in case surveillance for quick control measures.

## Conclusion

Digital technology has the potential to be developed during the COVID-19 pandemic, specifically in conducting data collection, surveillance, reporting, monitoring, and evaluation. Attitudes towards dengue control directly affect the perception of digital technology needs and program benefits. Social media is a more dominant source of information about dengue disease than other forms of electronic media. The perception of environmental factors is also directly influenced by the variables of threatened perception of dengue, program constraints, program benefits, and digital technology needs.

## Data Availability

Dryad: Attitude towards dengue control efforts with the potential of digital technology during COVID-19: partial least squares-structural equation modelling, https://doi.org/10.5061/dryad.jdfn2z3f0. ^38^ Data are available under the terms of the Creative Commons Zero “No rights reserved” data waiver (CC0 1.0 Public domain dedication). Figshare: Dengue integrated surveillance system questionnaire, https://doi.org/10.6084/m9.figshare.21300309. ^15^ Data are available under the terms of the Creative Commons Attribution 4.0 International license (CC-BY 4.0).
