# Supplementary material for: Attitude towards dengue control efforts with the potential of digital technology during COVID-19: partial least squares-structural equation modeling
Source: F1000Res. 2023 Jun 27;11:1283. Originally published 2022 Nov 10. [Version 2] doi: 10.12688/f1000research.125318.2 (PMC10333779; doi:10.12688/f1000research.125318.2)
Supplement: Peer review history

# Peer review: Reviewer response for version 2

**DOI:** 10.5256/f1000research.149494.r181880

**Published:** 2023-07-10

**Authors:** Mohammad Khaleel Okour

**Affiliations:** 1 Business Administration Department, Philadelphia University, Amman, Jordan

All required amendments have been made successfully. Thank you. I enjoyed reading this research.

Is the work clearly and accurately presented and does it cite the current literature?

Partly

If applicable, is the statistical analysis and its interpretation appropriate?

Partly

Are all the source data underlying the results available to ensure full reproducibility?

No source data required

Is the study design appropriate and is the work technically sound?

Yes

Are the conclusions drawn adequately supported by the results?

Partly

Are sufficient details of methods and analysis provided to allow replication by others?

Partly

Reviewer Expertise:

Technology Diffusion, Knowledge Management, Innovation Management

I confirm that I have read this submission and believe that I have an appropriate level of expertise to confirm that it is of an acceptable scientific standard.

# Peer review: Reviewer response for version 2

**DOI:** 10.5256/f1000research.149494.r181881

**Published:** 2023-07-04

**Authors:** Ahmad Firdhaus Arham

**Affiliations:** 1 Pusat Pengajian Citra Universiti, Bangi, Malaysia

All is good. Excellent.

Is the work clearly and accurately presented and does it cite the current literature?

Partly

If applicable, is the statistical analysis and its interpretation appropriate?

Partly

Are all the source data underlying the results available to ensure full reproducibility?

Yes

Is the study design appropriate and is the work technically sound?

Yes

Are the conclusions drawn adequately supported by the results?

Partly

Are sufficient details of methods and analysis provided to allow replication by others?

Yes

Reviewer Expertise:

Consumer Behavior on Contemporary Science, Tehnology and Sustainable Development Issues, SPSS and PLS-SEM.

I confirm that I have read this submission and believe that I have an appropriate level of expertise to confirm that it is of an acceptable scientific standard.

# Peer review: Reviewer response for version 1

**DOI:** 10.5256/f1000research.137611.r170731

**Published:** 2023-05-19

**Authors:** Ahmad Firdhaus Arham

**Affiliations:** 1 Pusat Pengajian Citra Universiti, Bangi, Malaysia

Introduction

- Lacks information about digital technology.
- What is the real problem statement? Why was this study needed, please relate strong problem statement.

Methodology

- Please bring out the variables section in your methodology. Suggest putting in the Theoretical Framework after introduction.
- Not Proper, to explain variables in your method, if you want to explain how the the variables and the items develop should be ok.

Analysis and result

- Please clarify your model fit or not - please test it.

Discussion

- Please focus on your results. What is your major finding in the first paragraph?

Overall, technically sounds ok.

Is the work clearly and accurately presented and does it cite the current literature?

Partly

If applicable, is the statistical analysis and its interpretation appropriate?

Partly

Are all the source data underlying the results available to ensure full reproducibility?

Yes

Is the study design appropriate and is the work technically sound?

Yes

Are the conclusions drawn adequately supported by the results?

Partly

Are sufficient details of methods and analysis provided to allow replication by others?

Yes

Reviewer Expertise:

Consumer Behavior on Contemporary Science, Tehnology and Sustainable Development Issues, SPSS and PLS-SEM.

I confirm that I have read this submission and believe that I have an appropriate level of expertise to confirm that it is of an acceptable scientific standard, however I have significant reservations, as outlined above.

# Response

**Published:** 2023-05-28

**Authors:** Dewi Susanna

**Affiliations:** Universitas Indonesia, Indonesia

Thank you very much for giving any comment for improvements. Here, we tried to respond your comments as follow:

**Introduction **

1. Lacks information about digital technology.

*Response:*

 The research objectives have been adjusted to:

 This study aims to identify critical indicators influencing attitudes towards dengue control related to the potential for implementing digital technology.

 Digital technology has developed rapidly in the health sector. Digital technology is beneficial during the Covid-19 pandemic for conducting dengue surveillance. The existence of a social restriction policy with social distancing has caused the door-to-door control program not to be implemented [11], [12], [13].

1. What is the real problem statement? Why was this study needed, please relate strong problem statement.

*Responses:*

 Attitudes in dengue control are essential because they can influence control measures. This model is needed in analyzing variables related to attitudes and the use of digital applications in conducting dengue surveillance. This study will obtain a potential model that can be used as a digital innovation for dengue control. This study aims to identify critical indicators influencing attitudes towards DHF control related to the potential for implementing digital technology.

**Methodology **

1. Please bring out the variables section in your methodology. Suggest putting in the Theoretical Framework after introduction.

*Response:*

 We have separated the Theoretical Framework after introduction. We have also explained in detail about the conceptual model and hypothesis before the method.

  

1. Not Proper, to explain variables in your method, if you want to explain how the the variables and the items develop should be ok.

*Responses:*

 In the partial least square-structural equation modelling (PLS-SEM) method, explaining the variables being analyzed is essential. This explanation facilitates the reader's understanding of the definition of these variables. The symbol of each variable needs to be defined so that it has the same meaning. It has been included in the research method.

**Analysis and result **

1. Please clarify your model fit or not - please test it.

*Responses:*

 An explanation of the structural model fit has been given in Table 6.

 The structural model is evaluated after verifying the correctness of the construction measurements. The path coefficients and their 10,000 resampling bootstrap significance levels are reported in Table 6 and Figure 6. Additionally, Table 6 shows that the VIF constructs range from 1,000 to 1,700, indicating no collinearity between variables. This study also assesses the quality by examining the overall predictive relevance of the model with a Q2 value above zero which indicates a fit in the prediction model. The magnitude of Q2 has a value of 0 < Q2 < 1, where the closer to 1, the better the model. The coefficient of determination (R2) also exceeds 0.1 for endogenous latent variables since the construct has an acceptable predictive power quality.

**Discussion**

 1. Please focus on your results. What is your major finding in the first paragraph?

*Responses:*

 A discussion of results and discussion has been added, as described below:

 This study aimed to determine the variables that influence attitudes in dengue control related to the potential application of digital technology. It indicates a direct influence of Perception of Environmental Factors on Threatened Perception of Dengue, Program Constraints, Program Benefits, and Digital Technology needs. Perception of Program Benefits and Threatened Perception of Dengue directly Influences Digital Technology Need. Perception of Digital Technology Needs and Program Benefits directly influence Attitude Towards Dengue Control.

 Dengue is still a public health problem in Asia, especially in tropical countries like Indonesia. Even during the COVID-19 pandemic, dengue became a double disease burden ^46^. During the COVID-19 pandemic, there was an increase in dengue infection, while dengue monitoring and control activities were limited in several countries ^47^. This condition poses a severe threat to dengue-endemic areas.

 Several studies have shown that dengue control measures are essential. Empowering the community to carry out activities to control water containers where mosquitoes breed is effective in preventing dengue infection ^48^, ^49^, ^50^. The Aedes aegypti mosquito as the primary vector needs to be eliminated ^51^. This attitude in controlling dengue is the primary key to preventing dengue outbreaks ^52^.

 Digital health surveillance technologies assist in disease prevention, detection, tracking, reporting and analysis ^53^, ^54^. The development of digital technology supported by the infrastructure can assist in reporting. An integrated digital surveillance system is needed for dengue control.

 The variable perception of the need for digital technology and program benefits directly influences attitudes toward dengue control. This is related to the source of information obtained through digital media. Perception of environmental factors is influenced by Threatened Perception of Dengue, Program Constraints, Program benefits, and Digital Technology Needs.

1. Overall, technically sounds ok.

*Response:*

 Thank you very much for your appreciation.

 Overall, thank you for all the input so this article can be even better.

 Regards,

 Tim.

# Peer review: Reviewer response for version 1

**DOI:** 10.5256/f1000research.137611.r156148

**Published:** 2022-12-05

**Authors:** Mohammad Khaleel Okour

**Affiliations:** 1 Business Administration Department, Philadelphia University, Amman, Jordan

I appreciate the effort that the authors did in this manuscript. However, I have some apprehensions which I have highlighted below:

**Introduction**

- The authors mainly explained the lack of understanding of the factors that influence attitudes toward dengue control concerning the potential application of digital technology in Indonesia. It will be more beneficial if they could provide further evidences based on previous studies from the context of Southeast Asia countries; specifically, Indonesian context.

**Literature review**

- I could not find any serious attempt to write a literature review related to the variables of the study. Researchers should create a new separate section that discuss the previous conducted studies in this field of research (based on the study model).

**Method**

- As the study is deductive in nature, the authors should clarify the rationale behind adopting the health belief theory. In its current form, there is not enough theoretical justification to propose the study hypotheses.
- The authors need to make sufficient justification on how they determined the sample size?
- A standard PLS-SEM methodology was utilized. More evidence should be provided that the statistical assumptions of PLS-SEM have been fulfilled before using it.
- In term of the study respondent characteristics, were there no respondents with master's or doctoral degrees?
- The authors must write the hypotheses of the study clearly and explicitly. For better readability, I recommend the authors create a new table that includes the hypothesis statement with its result (supported / not supported). 

**Discussion**

- Based on the study model, I don’t see a significant attempt to discuss the study findings. Authors must provide a detailed discussion that includes sufficient linkage/justification between the study findings with previous recent studies.

**Implication **

- The authors have focused on the practical/ managerial implications of the study. On the other hand, the authors have not given explicit discussion/ examples of theoretical implications.

Is the work clearly and accurately presented and does it cite the current literature?

Partly

If applicable, is the statistical analysis and its interpretation appropriate?

Partly

Are all the source data underlying the results available to ensure full reproducibility?

No source data required

Is the study design appropriate and is the work technically sound?

Yes

Are the conclusions drawn adequately supported by the results?

Partly

Are sufficient details of methods and analysis provided to allow replication by others?

Partly

Reviewer Expertise:

Technology Diffusion, Knowledge Management, Innovation Management

I confirm that I have read this submission and believe that I have an appropriate level of expertise to confirm that it is of an acceptable scientific standard, however I have significant reservations, as outlined above.

# Response

**Published:** 2023-05-28

**Authors:** Dewi Susanna

**Affiliations:** Universitas Indonesia, Indonesia

Thank you very much for giving any comments of our manuscript.

 We are very sorry for delaying posting these responses since we are highly recommended to wait for another reviewer’s report.

 We have discussed it to make our manuscript more understandable for readers.

 Here are our responses to all comments:

**Introduction**

*Question:*

 The authors mainly explained the lack of understanding of the factors that influence attitudes toward dengue control concerning the potential application of digital technology in Indonesia. It will be more beneficial if they could provide further evidences based on previous studies from the context of Southeast Asia countries; specifically, Indonesian context.

*Response:*

*Additional information has been added in the text. An explanation of this has been included in the background*

 During the COVID-19 pandemic, efforts to control dengue were hampered due to the limited door-to-door educational activities. Therefore, the use of digital technology can help carry out surveillance and education to the public. There are several studies in Indonesia regarding attitudes toward dengue control. A study in Kupang, Indonesia, explains that a significant relationship exists between knowledge, attitudes, and actions in controlling dengue ^11^. Likewise, other studies in Indonesia show that knowledge and attitudes influence dengue prevention measures ^12,13^. Another study in Indonesia on community perspectives on electronic-based dengue vector surveillance during the COVID-19 pandemic ^14^. There are also studies on developing a mobile-based dengue surveillance information system as an early warning system ^15,16^.

 Digital technology has developed rapidly in the health sector. Digital technology is beneficial during the Covid-19 pandemic for conducting dengue surveillance. The existence of a social restriction policy with social distancing has caused the door-to-door control program not to be implemented ^17, 18, 19^.

 Attitudes in dengue control are essential because they can influence control measures. This model is needed in analyzing variables related to attitudes and the use of digital applications in conducting dengue surveillance. This study will obtain a potential model that can be used as a digital innovation for dengue control. This study aimed to identify critical indicators influencing attitudes towards DHF control related to the potential for implementing digital technology.

**Literature review**

*Question*:

 I could not find any serious attempt to write a literature review related to the variables of the study. Researchers should create a new separate section that discuss the previous conducted studies in this field of research (based on the study model).

*Responses:*

*The Literature Review has been written in the paper as below:*

**Literature review**

 The co-epidemic trend of COVID-19 and dengue in Southeast Asia needs serious attention. These two diseases have similar clinical symptoms ^20, 21^. The COVID-19 pandemic situation is a challenge in controlling dengue in Indonesia. The existence of a social restriction policy makes it difficult for volunteers to provide education manually door to door. The impact is increased dengue cases in several areas ^22^.

 Public awareness regarding dengue control efforts still needs to be improved. Knowledge related to dengue can influence attitudes toward dengue control ^23, 24^. A positive attitude can encourage action to control dengue ^25, 26^. A large number of water containers is a breeding ground for mosquitoes. This is a potential mosquito breeding site that needs to be controlled. Several studies also state that interventions are needed to increase knowledge, attitudes, and actions in controlling dengue ^27, 28, 29^. Perceptions of the threat to the disease and the benefits of the interventions also influence attitudes towards dengue control. This is in accordance with the concept of the health belief model ^30^.

 Attitudes towards dengue control using digital technology need to be studied for the driving and inhibiting factors. Intervention with digital technology requires supporting facilities and infrastructure ^18, 17^. Digital educational media that can increase public knowledge is needed during the COVID-19 pandemic. Through socialization with digital educational media, prevention activities can be carried out widely despite a social distancing policy.

 The development of digital technology has helped health services to provide fast and integrated services. Particularly in dengue control, digital interventions can help conduct surveillance of areas with mosquito density, larval density, and several dengue cases so that they can make a priority scale ^31, 32^. Utilization of digital technology in conducting digital data collection and education can provide early detection and response to dengue cases in an area.

**Method**

 Question:

 As the study is deductive in nature, the authors should clarify the rationale behind adopting the health belief theory. In its current form, there is not enough theoretical justification to propose the study hypotheses.

*Response:*

 Conceptual model and hypotheses

 The theoretical model adopts a health belief model between perceptions and dengue control behaviour. ^11^^ –^^13^ The health belief theory is then modified by adding environmental variables and the need for digital technology. The hypotheses were compared with six latent constructs related to dengue control attitudes, influenced by perceptions of the threat of dengue, program benefits, environmental factors, program constraints, and technology needs (Figure 1). The direction of the path shows the (+) and (-) effects of the relationship, and this study assessed the accuracy of the model and hypothesis with PLS-SEM.

 Various factors influence attitudes in efforts to control dengue. During the COVID-19 pandemic, social restrictions were carried out ^36^. Social restrictions impact dengue control programs. Based on the theory of behavior change that a person is motivated to make prevention efforts when they feel seriously threatened and feel the benefits of intervention. The use of digital technology in dengue surveillance is also encouraged by the benefits of these digital applications and their ease of use. The health belief model approach is used because it is appropriate to form a model for changing attitudes towards dengue control related to perceptions of dengue threat, benefits of dengue control programs, and perceptions of constraints ^37, 38^.

 Environmental factors affect the transmission of dengue infection. Dengue is transmitted by the Aedes aegypti mosquito, which breeds in water containers. The large number of water containers in an environment can affect the density of mosquitoes. Climatic factors (temperature, humidity and rainfall), as well as population density, also increase dengue transmission ^39, 40^. Environmental factors can increase the potential for dengue transmission in an area. The perception of environmental factors is essential to analyze.

 Perceived ease of use and perceived benefits influence the perceived need for technology. This is in accordance with the Technology Acceptance Model (TAM) ^41^. Several studies on TAM are related to public acceptance of an application ^42, 43, 44, 45^. The use of dengue surveillance applications is needed to collect data and education. Digital applications can record quickly and integrate. This study uses a combination of several theories to create a suitable model according to stakeholder needs. The determinants of these variables are by the need to develop a dengue control attitude model related to technological needs and perceptions of environmental factors.

*Question:*

 The authors need to make sufficient justification on how they determined the sample size?

*Response:*

*The calculation of minimum sample was calculated using a formulae and added it in the text as follow*:

 The online survey was chosen because it was appropriate to do it during the Covid-19 pandemic. Online surveys are easier and cheaper than manual surveys using door to door.

 Measurement of sample size using the following formulation:

 S = Z ^2^ × P × (1−P)/M ^2^

 S = (1.960) ^2 ^× 0.5 ^^× (1−0.5)/0.05 ^2 ^= 3.8416 × 0.25 / 0.0025

 S= 384.16

 Notes:

 S = Sample size for infinite population

 P = Population proportion ( Assumed as 50% or 0.5)

 M = Margin of error = 5%

 Z = The Z-score will be 1.96 if the confidence level is 95%

 This means that: n = 384, z = 1.96, M = 0.05 and p = 0.5

*Question:*

 A standard PLS-SEM methodology was utilized. More evidence should be provided that the statistical assumptions of PLS-SEM have been fulfilled before using it.

*Response:*

 Measurement Model

 Composite Mode A

 The composite measurement model in mode A (attitude) was assessed in individual item reliability, construct reliability, convergent validity, and discriminant validity. First, the reliability of each item is analyzed through a loading factor, as seen in Figure 5.

*Question:*

 In term of the study respondent characteristics, were there no respondents with master's or doctoral degrees?

*Response:*

 The selected respondents were the general public who were willing to be involved in the Denpasar area, Bali. The results of the survey showed that there were no respondents with master's and doctoral degrees.

*Question:*

 The authors must write the hypotheses of the study clearly and explicitly. For better readability, I recommend the authors create a new table that includes the hypothesis statement with its result (supported / not supported).

*Response:*

 Based on the literature discussed, the following hypotheses emerge to provide the scope of this study:

 Hypothesis 1 (H1). Perceptions of environmental factors have a positive effect on perceptions of dengue threat.

 Hypothesis 2 (H2). Perceptions of environmental factors have a positive effect on perceptions of program constraints.

 Hypothesis 3 (H3). The perception of the dengue threat positively affects the perception of the need for digital technology.

 Hypothesis 4 (H4). Perceptions of environmental factors have a positive effect on perceptions of digital technology needs.

 Hypothesis 5 (H5). Perception of dengue threat has a positive effect on perceptions of program benefits.

 Hypothesis 6 (H6). Perceptions of program constraints positively affect perceptions of digital technology needs.

 Hypothesis 7 (H7). Perceived program constraints have a positive effect on attitudes toward dengue control.

 Hypothesis 8 (H8). Perception of being threatened by dengue positively affects attitudes toward dengue control.

 Hypothesis 9 (H9). Perceived benefits of the program positively affect attitudes toward dengue control.

 Hypothesis 10 (H10). Perception of the need for digital technology positively affects attitudes toward dengue control.

 Hypothesis 11 (H11). Perceptions of program benefits positively affect perceptions of digital technology needs.

**Discussion**

*Question*:

 Based on the study model, I don’t see a significant attempt to discuss the study findings. Authors must provide a detailed discussion that includes sufficient linkage/justification between the study findings with previous recent studies.

*Response:*

 Several research results have been added in the text.

 “This study aimed to determine the variables that influence attitudes in dengue control related to the potential application of digital technology. It indicates a direct influence of Perception of Environmental Factors on Threatened Perception of Dengue, Program Constraints, Program Benefits, and Digital Technology needs. Perception of Program Benefits and Threatened Perception of Dengue directly Influences Digital Technology Need. Perception of Digital Technology Needs and Program Benefits directly influence Attitude Towards Dengue Control.

 Dengue is still a public health problem in Asia, especially in tropical countries like Indonesia. Even during the COVID-19 pandemic, dengue became a double disease burden ^46^. During the COVID-19 pandemic, there was an increase in dengue infection, while dengue monitoring and control activities were limited in several countries ^47^. This condition poses a severe threat to dengue-endemic areas.

 Several studies have shown that dengue control measures are essential. Empowering the community to carry out activities to control water containers where mosquitoes breed is effective in preventing dengue infection ^48, 49, 50^. The Aedes aegypti mosquito as the primary vector needs to be eliminated ^51^. This attitude in controlling dengue is the primary key to preventing dengue outbreaks ^52^.

 Digital health surveillance technologies assist in disease prevention, detection, tracking, reporting and analysis ^53, 54^. The development of digital technology supported by the infrastructure can assist in reporting. An integrated digital surveillance system is needed for dengue control.

 The variable perception of the need for digital technology and program benefits directly influences attitudes toward dengue control. This is related to the source of information obtained through digital media. Perception of environmental factors is influenced by Threatened Perception of Dengue, Program Constraints, Program benefits, and Digital Technology Needs.”

**Implication**

*Question:*

 The authors have focused on the practical/ managerial implications of the study. On the other hand, the authors have not given explicit discussion/ examples of theoretical implications.

*Response*:

*Thank you for your input.* *For theoretical benefits, we have added that through this study a model for the development of science regarding attitude factors in controlling dengue with digital technology has been found.* *Quotations have been included in the article*:

 “This study adds to the literature and provides a comprehensive understanding related to attitudes in dengue control, perceptions of program benefits, perceptions of dengue threats, perceptions of constraints, perceptions of the need for digital technology, and perceptions of environmental factors ^38^^,^^71^. This study also contributes to supporting the health belief model ^30^^,^^72^. This study has added to the theoretical literature by developing a structural model related to dengue control attitudes, especially in Indonesia.”

 Overall, thank you for all the input so this article can be even better.

 Regards,

 Tim.
